# Hybrid Technologies for Reconstruction of Proximal Aortic Dissection

**DOI:** 10.17691/stm2023.15.3.05

**Published:** 2023-05-28

**Authors:** D.A. Sirota, M.O. Zhulkov, D.S. Khvan, T. Caus, B.N. Kozlov, A.V. Protopopov, A.G. Makayev, A.V. Fomichev, Kh.A. Agayeva, A.K. Sabetov, V.L. Lukinov, A.G. Edemsky, A.M. Chernyavsky

**Affiliations:** Head of the Research Department of Surgery on Aorta, Coronary and Peripheral Arteries, Institute of Blood Circulation Pathology; Meshalkin National Medical Research Center of the Ministry of Health of the Russian Federation, 15 Rechkunovskaya St., Novosibirsk, 630055, Russia; Cardiovascular Surgeon, Department of Aorta and Coronary Artery Surgery; Meshalkin National Medical Research Center of the Ministry of Health of the Russian Federation, 15 Rechkunovskaya St., Novosibirsk, 630055, Russia;; Researcher, Research Department of Surgery on Aorta, Coronary and Peripheral Arteries, Institute of Blood Circulation Pathology; Meshalkin National Medical Research Center of the Ministry of Health of the Russian Federation, 15 Rechkunovskaya St., Novosibirsk, 630055, Russia; Cardiovascular Surgeon, Department of Aorta and Coronary Artery Surgery; Meshalkin National Medical Research Center of the Ministry of Health of the Russian Federation, 15 Rechkunovskaya St., Novosibirsk, 630055, Russia;; Senior Researcher, Research Department of Surgery on Aorta, Coronary and Peripheral Arteries, Institute of Blood Circulation Pathology; Meshalkin National Medical Research Center of the Ministry of Health of the Russian Federation, 15 Rechkunovskaya St., Novosibirsk, 630055, Russia; Cardiovascular Surgeon, Department of Aorta and Coronary Artery Surgery; Meshalkin National Medical Research Center of the Ministry of Health of the Russian Federation, 15 Rechkunovskaya St., Novosibirsk, 630055, Russia;; Cardiovascular Surgeon; University Hospital Amiens, Avenue René Laënnec, Salouël, Amiens, 80054, France;; Head of the Department of Cardiovascular Surgery; Cardiology Research Institute, Tomsk National Research Medical Center of the Russian Academy of Sciences, 111a Kievskaya St., Tomsk, 634012, Russia;; Resident, Cardiovascular Surgeon; Meshalkin National Medical Research Center of the Ministry of Health of the Russian Federation, 15 Rechkunovskaya St., Novosibirsk, 630055, Russia;; Resident, Cardiovascular Surgeon; Meshalkin National Medical Research Center of the Ministry of Health of the Russian Federation, 15 Rechkunovskaya St., Novosibirsk, 630055, Russia;; Research Department of Surgery on Aorta, Coronary and Peripheral Arteries, Institute of Blood Circulation Pathology; Meshalkin National Medical Research Center of the Ministry of Health of the Russian Federation, 15 Rechkunovskaya St., Novosibirsk, 630055, Russia; Cardiovascular Surgeon, Department of Aorta and Coronary Artery Surgery; Meshalkin National Medical Research Center of the Ministry of Health of the Russian Federation, 15 Rechkunovskaya St., Novosibirsk, 630055, Russia;; Research Department of Surgery on Aorta, Coronary and Peripheral Arteries, Institute of Blood Circulation Pathology; Meshalkin National Medical Research Center of the Ministry of Health of the Russian Federation, 15 Rechkunovskaya St., Novosibirsk, 630055, Russia;; Cardiovascular Surgeon, Department of Aorta and Coronary Artery Surgery; Meshalkin National Medical Research Center of the Ministry of Health of the Russian Federation, 15 Rechkunovskaya St., Novosibirsk, 630055, Russia;; Senior Researcher; Institute of Computational Mathematics and Mathematical Geophysics of the Siberian Branch of the Russian Academy of Sciences, 6 Academician Lavrentyev Avenue, Novosibirsk, 630090, Russia Head of the Laboratory of Numerical Analysis of Stochastic Differential Equations; Institute of Computational Mathematics and Mathematical Geophysics of the Siberian Branch of the Russian Academy of Sciences, 6 Academician Lavrentyev Avenue, Novosibirsk, 630090, Russia; Researcher, Research Department of Surgery on Aorta, Coronary and Peripheral Arteries, Institute of Blood Circulation Pathology; Meshalkin National Medical Research Center of the Ministry of Health of the Russian Federation, 15 Rechkunovskaya St., Novosibirsk, 630055, Russia; Cardiovascular Surgeon, Department of Aorta and Coronary Artery Surgery; Meshalkin National Medical Research Center of the Ministry of Health of the Russian Federation, 15 Rechkunovskaya St., Novosibirsk, 630055, Russia;; Professor, Correspondent Member of the Russian Academy of Sciences, General Director; Meshalkin National Medical Research Center of the Ministry of Health of the Russian Federation, 15 Rechkunovskaya St., Novosibirsk, 630055, Russia;

**Keywords:** frozen elephant trunk, aortic dissection, aortic arch, thoracic aorta, thrombosis, major bleeding, stents

## Abstract

**The aim of the study** is to evaluate the efficacy of various types of hybrid technology in compare to the classical repair of the aortic arch of type I aortic dissection treatment in the in-hospital period.

**Materials and Methods:**

A retrospective observational study has been conducted, the results of surgical treatment of 213 patients with DeBakey type I aortic dissection operated on within the period from 2001 to 2017 were compared. Patients were divided into three groups: in group 1, patients undergone a hemiarch type of aortic repair or the total arch replacement (n=121); in group 2, a hemiarch aortic reconstruction and implantation of bare metal stent was performed (n=55); in group 3, a frozen elephant trunk technique was used (n=37). Taking into consideration the retrospective character of the investigation and nonequivalence of the groups by separate characteristics, they were equalized to improve the reliability of the results using the PSM (propensity score matching) pseudorandomization method. As a result, three groups of comparison were formed which were equalized by the PSM method and called PSM 1, 2, and 3. The mortality and complication rate in the in-hospital period, as well as the frequency of false lumen thrombosis development depending on the treatment method, have been analyzed.

**Results:**

The mortality rate in the PSM 1 group was 15 patients: group 1 (standard technique) — 10 patients (9%), group 2 (uncoated stents) — 5 patients (11%). A significant difference was found in the number of major bleedings (group 1 — 8%, group 2 — 21%, p=0.031) and cases of bowel ischemia (group 1 — 1%, group 2 — 9%, p=0.028). Complete false lumen thrombosis of the thoracic aorta was observed significantly more often in group 1 than in group 2 (22% vs 5%, p=0.015).

In the examined group PSM 2, hospital mortality rate was 4 patients: group 1 — 3 patients (12%), group 3 — 1 patient (3%). No differences between the groups were found in the number of complications. In group 3, complete false lumen thrombosis of the thoracic aorta was observed in 59% of cases, whereas in group 1 it was found only in 4% of patients (p<0.001).

In comparison group PSM 3, the mortality was 8 patients: group 2 — 5 patients (11%), group 3 — 3 patients (9%). The number of neurological complications differed significantly: in group 2 — 27%, in group 3 — 6% (p=0.019). Besides, 3% of cases of complete false lumen thrombosis were found in group 2, while there appeared 55% (p<0.001) of such patients in group 3.

**Conclusion:**

The comparative analysis showed that the use of bare metal stents and hybrid prostheses demonstrated a comparable low level of in-hospital mortality compared to the standard surgical technique of aortic arch reconstruction. At the same time, the use of the bare metal stents is associated with a higher rate of perioperative complications (bleeding, postoperative bowel ischemia, neurological complications) compared to the standard treatment and repair of the aortic dissection using hybrid prostheses. Complete thrombosis of the false lumen occurred significantly less commonly in case of using bare metal stents than with standard treatment and hybrid prostheses.

## Introduction

Aortic dissection remains a serious problem in modern cardiac surgery and is associated with a high mortality rate in the early and long-term postoperative period. According to studies, the morbidity varies from 2.9 to 6.0 cases per 100 thousand population per year [1-3]. The peak age for Stanford type A dissection is 50–60 years, whereas type B dissection is 60–70 years [4, 5]. Indications for surgical treatment in patients with these diseases are absolute [6, 7]. The mortality rate in the natural history of type A acute dissection is extremely high. Thus, according to Genoni et al. [8], approximately 20% of patients die within the first hours of the event before hospitalization. Without surgical treatment, the mortality rate amounts to 25% in the first 6 h and reaches 50% by the end of the first day; by the end of the first week without treatment, approximately 75% of patients die [8, 9]. Despite the improvement in surgical technique, anesthetic support, technical and technological provision, in-hospital mortality remains high. According to the data of the International Registry of Acute Aortic Dissection (IRAD), the mortality rate for conservative treatment of acute type A dissection is 58%, compared with 26% for surgical treatment [10, 11].

The main, and in some cases the only way of treating aortic dissection is its open reconstruction [12, 13]. According to the existing recommendations, surgical intervention on the dissected ascending aorta must obligatorily include resection of the primary intimal tear and replacement of the ascending aorta. The level of the distal anastomosis is an essential aspect in the surgical management of proximal dissections and depends on the way the aortic wall dissection is spreading, presence of tears, fenestrations, thrombosis in the arch, as well as involvement of brachiocephalic vessels. Some authors [14-16] recommend expanding the resection volume and performing total replacement of the arch as often as possible reasoning that this kind of intervention improves remote results due to the absence of the blood flow in the false lumen, whereas manipulations on the distal aorta have a high risk of mortality and complications. However, according to the other authors, the hemiarch repair demonstrates similar results [17].

The mortality rate for conservative treatment of acute type B dissection is 10%, whereas in case of surgical intervention it grows up to 30% [4]. At present, with the gradual improvement of the immediate results of aortic dissection treatment, the long-term results depend to a great extent, on the presence of the patent false lumen, which is a predictor of reinterventions and fatal outcomes [18]. To increase the radicality, it has become technically feasible to treat aortic dissection by implanting ancillary devices simultaneously with the classical (traditional) intervention, which is usually performed by a hemiarch approach or aortic arch. However, the efficacy of the adjunctive aortic stenting simultaneously with the reconstructive operations on the aorta has not been well studied [19].

**The aim of the study** is to evaluate the efficacy of various types of hybrid technology in compare to the classical repair of the aortic arch of type I aortic dissection treatment in the in-hospital period.

## Materials and Methods

A retrospective observational study has been conducted, in which the results of surgical treatment of 213 patients with DeBakey type I aortic dissection were compared. The ascending aorta, aortic arch, and descending thoracic aorta were involved in all patients. The patients were operated on at the clinics of E.N. Meshalkin National Medical Research Center (Novosibirsk, Russia), Cardiology Research Institute of the Tomsk National Medical Center of the Russian Academy of Sciences (Tomsk, Russia), and University Hospital Amiens (France) from 2001 to 2017 were participated in the study. Data on the period of in-hospital observation are given in the article. The study design is presented in the Figure.

**Figure:**
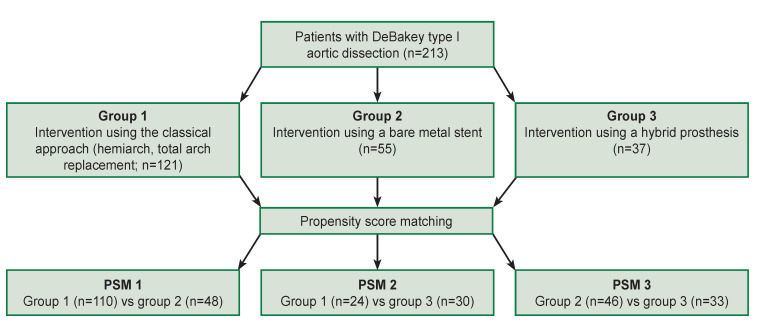
Study design

All participants were divided into 3 treatment groups: group 1 included patients undergone standard surgical approaches (hemiarch technique or total aortic arch replacement, 121 patients); group 2 — the hemiarch procedure or the total replacement of the aortic arch using bare metal stent (55 patients); group 3 undergone the frozen elephant trunk (FET) intervention (37 patients).

The groups were compared in pairs. All patients, included in the study, were confirmed the diagnosis in the preoperative period by ultrasound and tomographic investigations.

The most important aspects in making ultrasound and tomographic investigations are presented below:

1. To validate the diagnosis of aortic dissection by objective data (in contrast to the ultrasound methods).
2. To evaluate the condition of aortic root and valve, to define the degree and possible mechanism of aortic insufficiency as one of the most severe complications of proximal aortic dissection.
3. To assess the condition of aortic arch branches (blood flow preservation, dissection of the carotid and subclavian arteries) for determining the tactics of connecting cardiopulmonary bypass and the volume of intervention on the aortic arch.
4. To determine the condition of the thoracoabdominal aorta. To assess the presence and the false lumen expansion, to define the displacement of the visceral branch ostia (from the true and false lumen) and preservation of the blood flow in the organ arteries.
5. To define the presence or absence of secondary fenestrations of the aortic intima with the connection of the true and false lumens in the distal parts.

At discharge or after 30 days of hospitalization, all patients underwent control contrast-enhanced MSCT examination to test the aorta condition. Thrombosis of the false lumen was evaluated over the entire lumen length irrespective of the anatomical zone. Absence of the false lumen opacification indicated complete thrombosis, whereas absence of any evidence of false lumen thrombosis was considered to be complete patency.

Taking into consideration the retrospective character of the investigation and nonequivalence of the groups by separate characteristics, they were equalized to improve the reliability of the results using the PSM (propensity score matching) method of pseudorandomization. As a result, three groups of comparison were formed, equalized by the PSM method, and called PSM 1, 2, and 3. The lethality and complication rates in the in-hospital period depending on the examined group of comparison have been analyzed.

The nearest neighbor matching (NNM) method with a caliper value of 0.25 was applied to the initial groups to equalize the preoperative indices deliberately discarding inappropriate patients in the groups, with a given ratio of the desired groups 3:1 for groups 1 and 2 and 2:1 for other groups [20]. As a result, 110 and 48 selected patients were assigned to groups 1 and 2 (PSM 1), 24 and 30 patients to groups 1 and 3 (PSM 2), 46 and 34 patients to groups 2 and 3 (PSM 3). In the modified NNM method with caliper, the selection of the nearest neighbor in each individual was performed within the interval of the caliper size, the middle of the interval was taken equal to the individual’s score. When more than two nearest neighbors are selected, the sampling may happen to be incomplete and fewer neighbors may be selected for the individual than specified, which together with the preselection, leads to unstrictly proportional sizes of the selected groups [21]. The NNM method with caliper, unlike the conventional NNM method, allows for a more flexible of the matching and selection of a greater number of patients for the groups without sacrificing the quality of the matching. It should also be noted that the unequal number of patients in the comparison groups after the application of PSM was caused by the desire to maintain their maximum number, which is permissible since there was no difference between the examined groups studied before the operation.

### Statistical data processing

The compared continuous data of age, days from the event onset to surgery, weight, and height were tested for normality of distribution using the Shapiro-Wilk test and for equality of dispersion using the F-test. In the absence of normality, these parameters were compared using the nonparametric Mann–Whitney U test. The pseudomedian (PMe) of pairwise differences and the standardized mean difference (SMD) were calculated to assess differences between continuous parameters. The values of the continuous parameters were presented as a median, the first and third quartiles (Me [Q1; Q3]), mean and standard deviation (M±SD). The binary parameters: mortality, complications, and false lumen patency were described as the number of events, percentage of the total number of patients with construction of a 95% confidence interval according to the Wilson formula (n/%, 95% CI). Binary parameters were compared using Fisher’s bilateral exact test with the evaluation of the odds ratio (OR) and risk difference (RD). Differences were considered statistically significant at p<0.05.

All statistical computations were performed in the IDE RStudio (version 2022.07.2 build 576 ©2009–2022, PBC, USA) using the statistical computing R language (version 4.1.3, Austria).

## Results

The preoperative description of the comparison groups is presented in Table 1 for PSM 1, in Table 2 — for PSM 2, in Table 3 — for PSM 3. The tables show that all groups were statistically indistinguishable in the main characteristics.

**Table 1:** Preoperative description of the comparison group PSM 1

Variables	Group 1 (n=110)	Group 2 (n=48)	Difference (effect value)	p
Male gender:				
n/%	81/74	31/65	OR — 0.7	
95% CI	65–81	50–77	RD — 9%	0.259
Age (years):				
Me [Q1; Q3]	54 [45; 61]	58.0 [45.0; 65.25]	PMe — 2.0	
M±SD	54.17±14.14	55.44±15.3	SMD — 0.09	0.372
Days from the event to operation:				
Me [Q1; Q3]	52 [1; 291]	66.5 [3.5; 1122.0]	PMe — 16.9	
M±SD	96.06±975.32	1191.40±5889.18	SMD — 0.33	0.264
Weight (kg):				
Me [Q1; Q3]	79.50 [70.0; 89.75]	81.50 [75.0; 91.25]	PMe — 4.0	
M±SD	80.35±17.33	82.94±13.41	SMD — 0.16	0.101
Height (cm):				
Me [Q1; Q3]	172.50 [167.0; 178.75]	175 [170; 184]	PMe — 3.0	
M±SD	172.96±9.17	175.71±9.95	SMD — 0.29	0.079
Connective tissue diseases:				
n/%	21/19	9/19	OR — 1.0	
95% CI	13–27	10–32	RD — 0%	>0.999
Marfan syndrome:				
n/%	12/20	2/10	OR — 0.4	
95% CI	12–31	3–29	RD — 10%	0.502
Previous cardiac surgery:				
n/%	6/5	6/12	OR — 2.5	
95% CI	3–11	6–25	RD — 7%	0.188
Complicated aortic dissection:				
n/%	59/54	24/50	OR — 0.9	
95% CI	44–63	36–64	RD — 4%	0.730

**Table 2:** Preoperative description of comparison group PSM 2

Variables	Group 1 (n=24)	Group 3 (n=30)	Difference (effect value)	p
Male gender:				
n/%	17/71	16/53	OR — 0.5	
95% CI	51–85	36–70	RD — 18%	0.263
Age (years):				
Me [Q1; Q3]	45 [38; 52]	50.50 [46.25; 56.50]	PMe — 5.7	
M±SD	45.19±9.78	51.23±8.83	SMD — 0.64	0.084
Days from the event to operation:				
Me [Q1; Q3]	35 [6; 212]	141.0 [48.75; 754.50]	PMe — 106.0	
M±SD	422.0±1009.84	562.86±838.71	SMD — 0.15	0.087
Weight (kg):				
Me [Q1; Q3]	77.50 [61.25; 94.50]	74.0 [67.0; 88.25]	PMe — 0.98	
M±SD	79.41±22.74	79.83±19.33	SMD — 0.02	0.919
Height (cm):				
Me [Q1; Q3]	172.0 [166.50; 178.75]	172 [166; 176]	PMe — 1.0	
M±SD	172.64±8.48	171.59±8.88	SMD — 0.12	0.634
Connective tissue diseases:				
n/%	5/21	9/30	OR — 1.6	
95% CI	9–40	17–48	RD — 9%	0.540
Marfan syndrome:				
n/%	5/21	1/3	OR — 0.1	
95% CI	9–40	1–17	RD — 18%	0.078
Previous cardiac surgery:				
n/%	4/17	4/13	OR — 0.8	
95% CI	7–36	5–30	RD — 3%	>0.999
Complicated aortic dissection:				
n/%	12/50	9/30	OR — 0.4	
95% CI	31–69	17–48	RD — 20%	0.167

**Table 3:** Preoperative description of comparison group PSM 3

Variables	Group 2 (n=46)	Group 3 (n=34)	Difference (effect value)	p
Male gender:				
n/%	28/61	18/53	OR — 0.7	
95% CI	46–74	37–69	RD — 8%	0.502
Age (years):				
Me [Q1; Q3]	57.0 [44.25; 65.75]	50.50 [46.25; 56.50]	PMe — 4.5	
M±SD	54.76±15.73	51.23±8.83	SMD — 0.26	0.208
Days from the event to operation:				
Me [Q1; Q3]	89.0 [4.25; 1390.0]	141.0 [48.75; 754.50]	PMe — 63.0	
M±SD	1266.15±6003.12	562.86±838.71	SMD — 0.15	0.345
Weight (kg):				
Me [Q1; Q3]	81.0 [75.0; 91.75]	75.0 [67.25; 82.50]	PMe — 5.0	
M±SD	83.02±14.30	79.79±17.65	SMD — 0.2	0.097
Height (cm):				
Me [Q1; Q3]	175 [170; 184]	172 [164; 176]	PMe — 4.0	
M±SD	175.59±10.16	171.09±9.86	SMD — 0.45	0.070
Connective tissue diseases:				
n/%	9/20	13/38	OR — 2.5	
95% CI	11–33	24–55	RD — 19%	0.080
Marfan syndrome:				
n/%	2/10	1/3	OR — 0.3	
95% CI	3–29	1–15	RD — 7%	0.551
Previous cardiac surgery:				
n/%	8/17	5/15	OR — 0.8	
95% CI	9–31	6–30	RD — 3%	>0.999
Complicated aortic dissection:				
n/%	20/43	9/26	OR — 0.5	
95% CI	30–58	15–43	RD — 17%	0.159

The mortality rate in the ***PSM 1*** group was 15 patients: group 1 (standard technique) — 10 patients (9%), group 2 (bare stents) — 5 patients (11%) (Table 4).

**Table 4:** Complications, mortality rate, and false lumen status in the in-hospital period in patients of comparison group PSM 1

Variables	Group 1 (n=110)			Group 2 (n=48)			Fisher’s exact bilateral test, p
	Amount of data (n/%)	Number of cases (n/%)	95% CI	Amount of data (n/%)	Number of cases (n/%)	95% CI	
Major bleedings	110/100	9/8	4–15	47/98	10/21	12–35	0.031*
Neurological complications (all)	108/98	22/20	14–29	46/96	12/26	16–40	0.525
Myocardial infarction	109/99	4/4	1–9	47/98	4/9	3–20	0.243
Bowel ischemia	108/98	1/1	0–5	46/96	4/9	3–20	0.028*
In-hospital mortality	110/100	10/9	5–16	47/98	5/11	5–23	0.771
Complete thrombosis or obliteration	107/97	23/22	15–30	41/85	2/5	1–16	0.015*
Partial thrombosis	105/95	27/26	18–35	40/83	13/32	20–48	0.414
Completely patent false channel	106/96	56/53	43–62	41/85	26/63	48–76	0.271

* statistically significant difference between the variables.

The main causes of mortality in all comparison groups were acute cerebrovascular disease, myocardial infarction, and major bleeding. Major hemorrhage is defined as a condition requiring repeated surgery to remove the cause of the event.

When analyzing the frequency of postoperative complications, major bleeding and postoperative bowel ischemia were significantly more common in group 2 compared to group 1 (21% vs 8% and 9% vs 1%, respectively). CT scan before discharging showed that complete thrombosis of the false lumen was observed more often in group 1 than in group 2 (22% vs 5%).

The results of the postoperative period in the ***PSM 2*** group (groups 1 and 3) were similarly compared. The mortality was described in 4 patients: group 1 — 3 patients (12%), group 3 — 1 patient (3%) (Table 5).

**Table 5:** Complications, mortality rate, and false lumen status in the in-hospital period in patients of comparison group PSM 2

Variables	Group 1 (n=24)			Group 2 (n=30)			Fisher’s exact bilateral test, p
	Amount of data (n/%)	Number of cases (n/%)	95% CI	Amount of data (n/%)	Number of cases (n/%)	95% CI	
Major bleedings	24/100	4/17	7–36	29/97	8/28	15–46	0.512
Neurological complications (all)	23/96	4/17	7–37	29/97	2/7	2–22	0.387
Myocardial infarction	23/96	1/4	1–21	29/97	0/0	0–12	0.442
Bowel ischemia	23/96	1/4	1–21	29/97	3/10	4–26	0.621
In-hospital mortality	24/100	3/12	4–31	30/100	1/3	1–17	0.318
Complete thrombosis or obliteration	23/96	1/4	1–21	29/97	17/59	41–74	<0.001*
Partial thrombosis	23/96	9/39	22–59	29/97	12/41	26–59	>0.999
Completely patent false channel	23/96	13/57	37–74	27/90	0/0	0–12	<0.001*

* statistically significant differences between the variables.

Regarding the spectrum of complications in the comparison group PSM 2, no differences between the groups were found, despite the apparently more complicated intervention with the use of hybrid prostheses in this group. CT-scan angiography performed at discharge showed a high number of cases of complete false lumen thrombosis among patients of group 3 (59% vs 4% in group 1), with the preservation of the complete false lumen patency in 57% of cases in group 1 and its complete absence in group 3.

In the comparison group ***PSM 3*,** the lethality was 8 patients: group 2 — 5 patients (11%), group 3 — 3 patients (9%) (Table 6).

**Table 6:** Complications, mortality rate, and false lumen status in the in-hospital period in patients of comparison group PSM 3

Variables	Group 1 (n=46)			Group 2 (n=34)			Fisher’s exact bilateral test, p
	Amount of data (n/%)	Number of cases (n/%)	95% CI	Amount of data (n/%)	Number of cases (n/%)	95% CI	
Major bleedings	45/98	11/24	14–39	33/97	9/27	15–44	0.798
Neurological complications (all)	44/96	12/27	16–42	33/97	2/6	2–20	0.019*
Myocardial infarction	45/98	4/9	4–21	33/97	1/3	1–15	0.389
Bowel ischemia	45/98	4/9	4–21	33/97	4/12	5–27	0.718
In-hospital mortality	45/98	5/11	5–23	33/97	3/9	3–24	>0.999
Complete thrombosis or obliteration	39/85	1/3	0–13	33/97	18/55	38–70	<0.001*
Partial thrombosis	38/83	12/32	19–47	33/97	15/45	30–62	0.327
Complete patent false channel	39/85	26/67	51–79	31/91	0/0	0–11	<0.001*

* statistically significant differences between the variables.

When analyzing the frequency of perioperative complications, a statistically significant higher number of neurological complications were observed in the group of patients with uncoated stents (27% vs 6%, p=0.019). The comparative analysis of the CT data showed that significantly larger number of cases of complete false lumen thrombosis were detected in group 3 (55% vs 3% in group 2, p<0.001), and there were no cases of completely patent false lumen in group 3, whereas they were found in 67% (p<0.001) of patients in group 2.

## Discussion

According to the existing recommendations, surgical intervention on the ascending aorta due to its dissection must obligatorily include resection of the initial intimal tear followed by replacement [22]. However, there is no consensus on the need for the extensive intervention on the aortic arch and its descending part has not been reached so far [23, 24]. There are also no clear data on the effect of the technology used (bare metal stents or hybrid grafts) on the rate of the perioperative complications and mortality.

Our data show that the use of a balloon-expandable uncoated stent and a hybrid graft has a similar in-hospital mortality rate to the standard surgical technique of aortic arch reconstruction and is within 3–12% depending on the group studied. Aftab et al. [17] report that the in-hospital mortality rate for the standard approach was 29% in the hemiarch group and 22% in the total arch replacement. At the same time, the study by Kumagai and Minatoya [25] showed that the level of perioperative mortality is 4.5% in the hemiarch groups and 3.5% in the total arch replacement groups. Shrestha et al. [26] reported 12% mortality rate after the use of a multibranch Thoraflex hybrid prosthesis. After analyzing the results of the use of a Frozenix hybrid prosthesis, Uchida et al. [27] defined an in-hospital mortality rate of 5.0%, although it should be taken into consideration that 38 patients had thoracic aortic aneurysm and only 22 had aortic dissection.

Regarding the results of the use of uncoated stents in aortic dissection surgery, there are data obtained by Leobon et al. [28], who used Djumbodis stents in 22 patients with acute DeBakey type I aortic dissection. The 30-day mortality was 22.7%. Piccardo et al. [29] used the Djumbodis stent in 20 patients; the in-hospital mortality was 30%. A 30-day mortality in 1 of 6 patients (16.7%) was reported by Komarov et al. [30].

When analyzing the number of postoperative complications, we found that major bleeding (21% vs 8%, p=0.031) and postoperative bowel ischemia (9% vs 1%, p=0.028) were more frequently observed in balloon-expandable bare metal stent group compared to the standard treatment group, and there were more neurological events (27% vs. 6%, p=0.019) than in the hybrid prosthesis group. Hybrid aortic arch reconstruction did not increase the number of complications in the early postoperative period compared to standard technology. According to the data presented by Tsagakis and Jakob [31], 307 patients underwent thoracic aortic repair using the frozen elephant trunk technique with E-vita open and E-vita open plus hybrid stent-grafts. The overall 30-day mortality rate was 11.7% with cerebrovascular and spinal cord injuries occurring in 7.2 and 2.9% of cases, respectively, and major bleeding in 10.4% of cases. Shrestha et al. [26] reported that the number of strokes ranged from 10 to 18% in the group of chronic and acute dissection groups, respectively, and the rate of surgical hemostasis varied from 19.0 to 20.3%.

Since the success of the intervention both in the early and remote periods, is largely determined by the freedom from aortic-related events, false lumen patency as a predictor of adverse events in the postoperative period is the most important criterion for the efficacy of the proposed technologies. The authors of the study [28] consider the preservation of the false lumen patency to be a predictor of the repeated reintervention in the long-term period, therefore the surgical treatment strategy must be implemented in such a way as to maximally promote the obliteration of the false lumen in the descending aorta. In our series, despite the additional intervention, complete thrombosis of the false lumen was observed at hospital discharge more often in the standard treatment group than in the bare metal stent (22% vs 5%). The advantage in this parameter was expected in patients with a hybrid prosthesis. Thus, the results of the CT-scan angiography performed at discharge showed a higher number of cases of complete false lumen thrombosis among patients in group 3 — 59% vs 4% of cases in the standard treatment group. Compared to the group treated with uncoated stents, a significantly higher number of such cases was also found in group 3 — 55% vs 3% in group 2 (p<0.001), although there were no cases of completely patent false lumen in group 3, compared to 67% (p<0.001) of them detected in group 2.

Komarov et al. [30] reported 4 cases (66.7%) of partial thrombosis of the false lumen and only 1 case (16.7%) of complete thrombosis. In the series of 15 patients, Czerny et al. [32] observed 1 case (8%) of complete false lumen thrombosis and 4 (25%) cases of partial thrombosis, with no evidence of thrombosis were found in remaining patients.

### Study limitations

The retrospective study design imposes limitations on the representativeness and homogeneity of the sample, the propensity score matching method of pseudorandomization partially solves these problems. The short period of observation period is also a limitation of the study.

## Conclusion

The present retrospective study comparing the results of the surgical treatment of patients with DeBakey type I aortic dissection has shown that the use of bare-metal stent and a hybrid prosthesis does not significantly influence the in-hospital mortality rate, although there was a statistically significant increase in the number of perioperative complications in the bare metal stent group. The medium and long-term results require further observation and analysis, which will allow us to develop a personified approach to the treatment of each patient.
